# Supplementary material for: High-throughput fitness experiments reveal specific vulnerabilities of human-adapted Salmonella during stress and infection
Source: Nat Genet. 2024 Jun 3;56(6):1288–99. doi: 10.1038/s41588-024-01779-7 (PMC11176087; doi:10.1038/s41588-024-01779-7)
Supplement: Supplementary file 1 — Supplementary Figs. 1–16 and Notes 1 and 2. [file 41588_2024_1779_MOESM1_ESM.pdf]

nature genetics

Supplementary information https://doi.org/10.1038/s41588-024-01779-7

High-throughput fitness experiments reveal specific vulnerabilities of human-adapted *Salmonella* during stress and infection

In the format provided by the authors and unedited

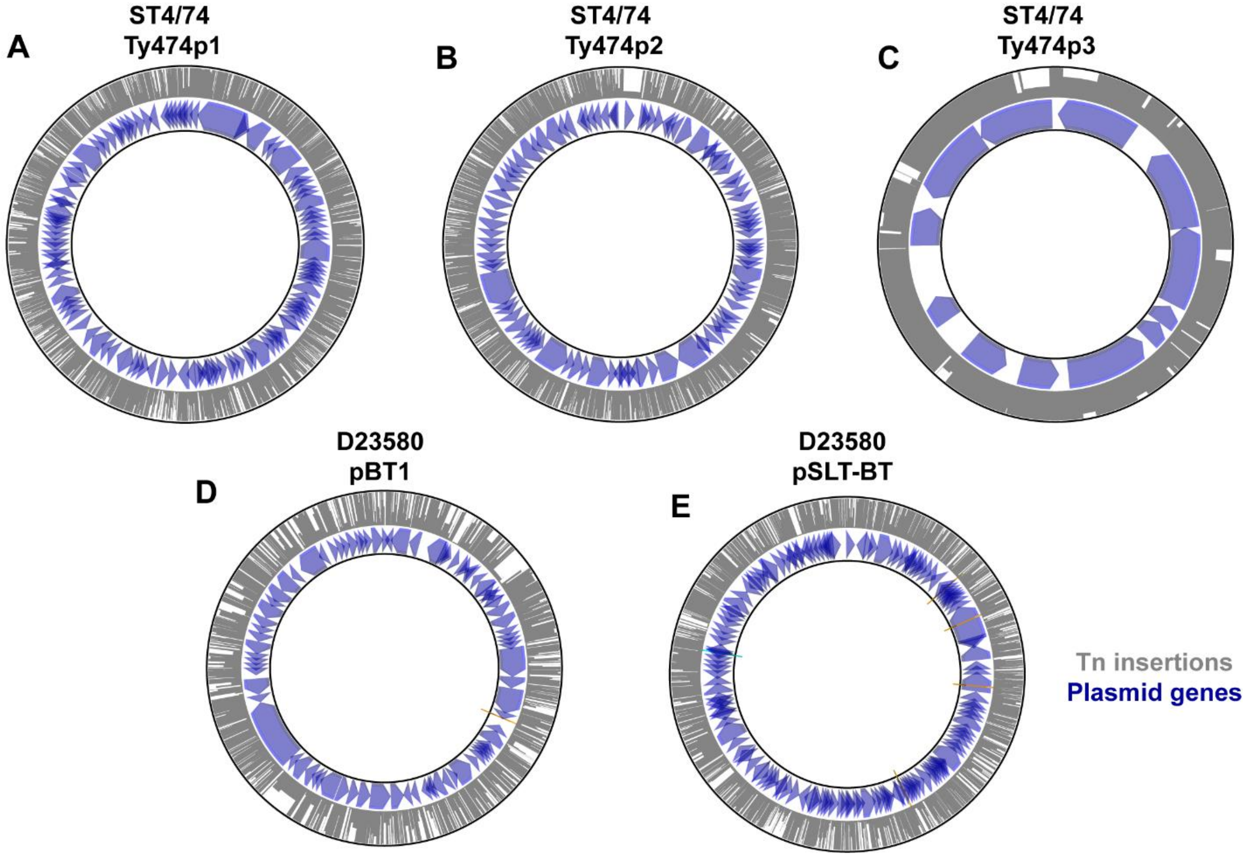

Supplemental Figure 1

Schematic of the location of each barcoded Tn insertion in the plasmids of Typhimurium ST4/74 and D23580. Inner circles containing blue arrows are the plasmid genes; each gene is denoted by an individual blue arrow. All Tn insertions are on the outer track in grey. Ty474p1 in ST4/74 is shown in **A,** Ty474P2in ST4/74 is shown in **B,** Ty474p3 in ST4/74 is shown in **C,** pBT1 in D23580 is shown in **D,** pSLT-BT in D23580 is shown in **E.**

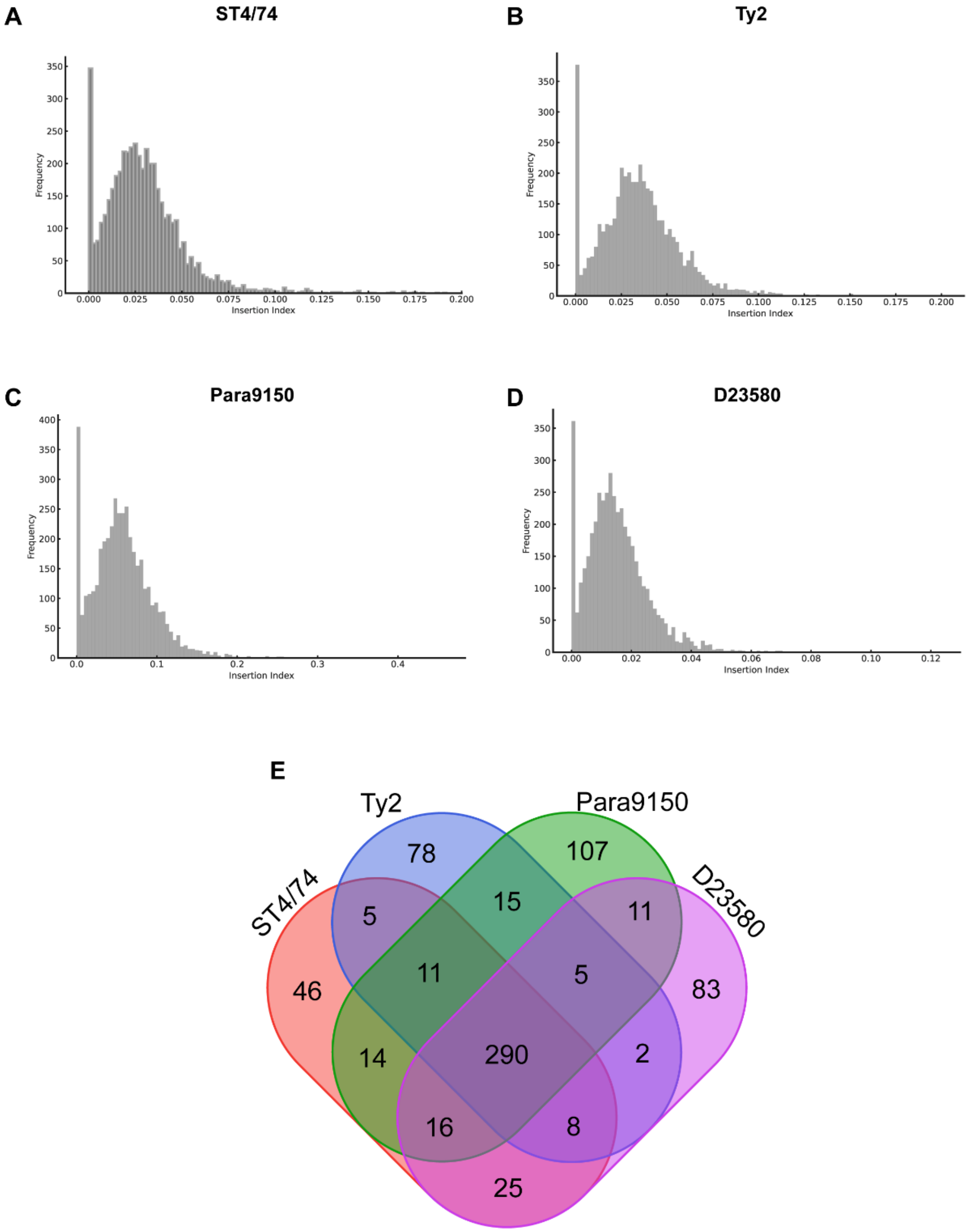

Supplemental Figure 2

Histogram of Tn insertion index (defined as # of unique Tn insertions divided by the length of the gene) in ST/474 (**A**)**,** Ty2 (**B**)**,** Para9150 **(C),** D23580 **(D).** A clear bimodal distribution can be seen, with one peak at zero (essential genes) and another peak greater than zero (non-essential genes). **E)** Venn diagram showing the shared and unique essential genes among the 4 isolates; ST4/74 in red, Ty2 in green, Para9150 in blue, and D23580 in purple.

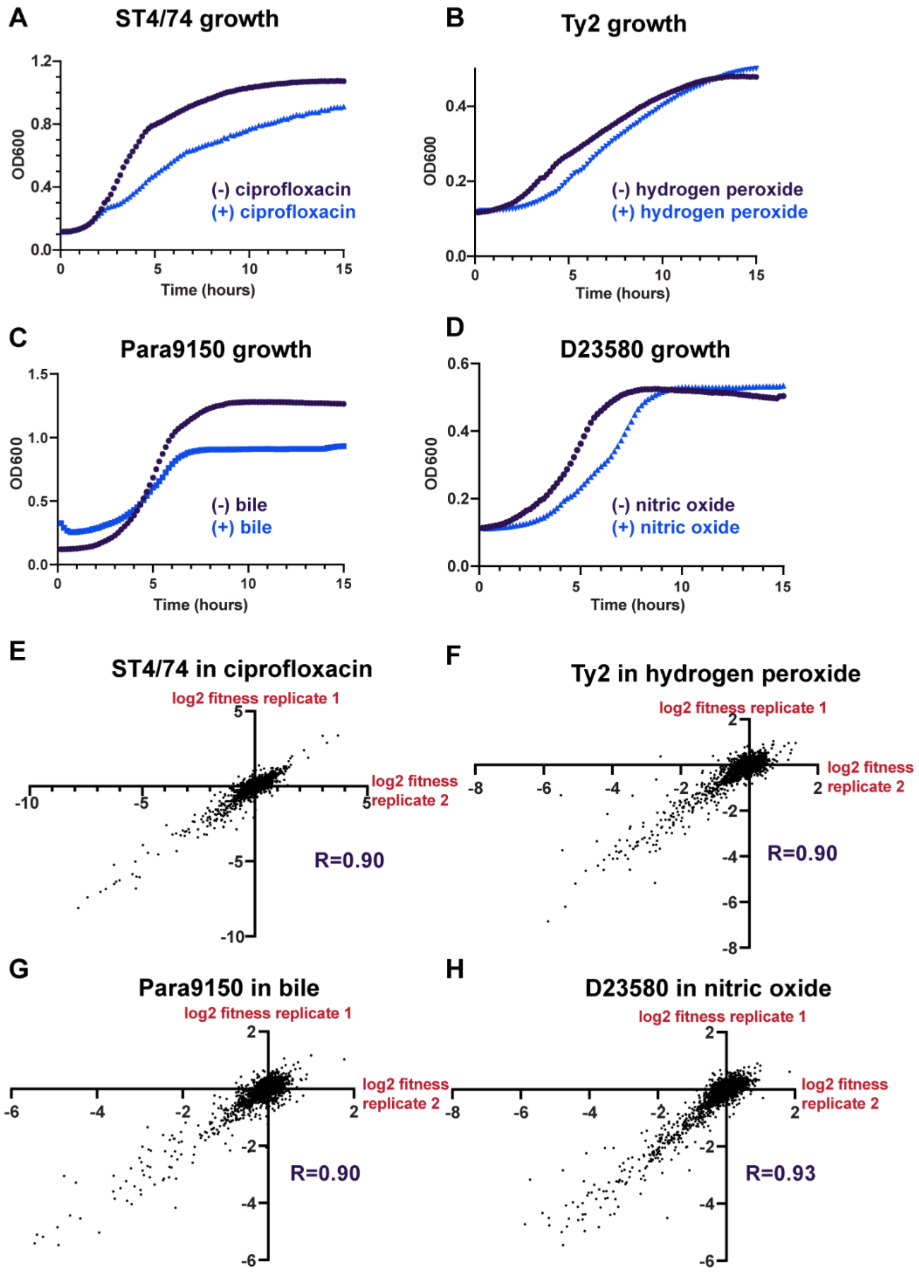

Supplemental Figure 3

A) Representative growth curve of *S.* Typhimurium ST4/74 grown in the absence (black) or presence (blue) of 0.0125 µg/mL ciprofloxacin, with reads taken at OD_{600} once every 10 minutes. **B)** Representative growth curve of *S.* Typhi Ty2 grown in the absence (black) or presence (blue) of 250 µM hydrogen peroxide, with reads taken at OD_{600} once every 10 minutes. **C)** Representative growth curve of *S.* Paratyphi A grown in the absence (black) or presence (blue)

of 4% ox bile, with reads taken at OD_{600} once every 10 minutes. **D)** Representative growth curve of *S.* Typhimurium D23580 grown in the absence (black) or presence (blue) of 1 mM nitric oxide, with reads taken at OD_{600} once every 10 minutes. **E)** Correlation between independent biological replicates of Rb-Tn-seq experiments performed with *S.* Typhimurium ST4/74 in the presence of 0.0125 µg/mL ciprofloxacin, where each dot represents the fitness of a gene. **F)** Correlation between independent biological replicates of Rb-Tn-seq experiments performed with *S.* Typhi Ty2 in the presence of 250 µM hydrogen peroxide, where each dot represents the fitness of a gene. **G)** Correlation between independent biological replicates of Rb-Tn-seq experiments performed with *S.* Paratyphi A 9150 in the presence of 4% ox bile, where each dot represents the fitness of a gene. **H)** Correlation between independent biological replicates of Rb-Tn-seq experiments performed with *S.* Typhimurium D23580 in the presence of 1 mM nitric oxide, where each dot represents the fitness of a gene. For all growth curves (**A-D**), each point and error bar indicates the mean ± SEM of OD_{600} with reads taken at OD_{600} once every 10 minutes. For all correlation plots (**E,F,G,H**), the correlation value (R) for these biological duplicates is shown in each plot.

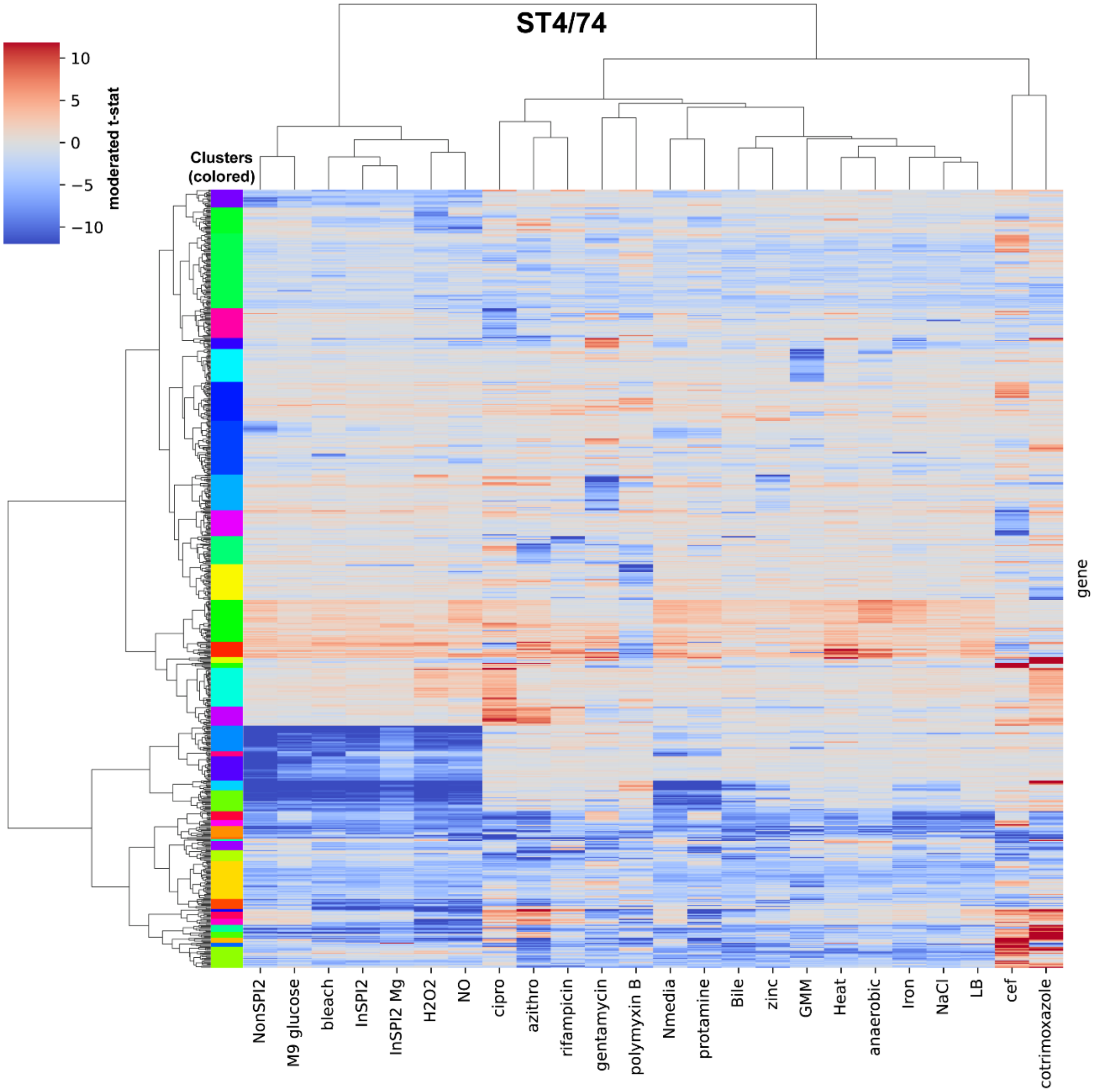

Supplemental Figure 4

Heatmap of genes with significant fitness effects (|moderated t-statistic|>4) from *S.* Typhimurium ST4/74, in which the moderated t-statistic from each gene is plotted across all 24 *in vitro* plate-based conditions. Each gene is an individual row, and each condition is an individual column. Pearson's correlation coefficient was used to calculate the similarity in fitness between pair of genes, and agglomerative clustering was used to cluster genes with more similar fitness effects. This hierarchical clustering was then visualized using dendrograms. Different clusters are highlighted in a series of colors to the left of the heatmap; a full list of genes in these clusters can be found in **Supplemental Data 5**). This heatmap was generated from the average of two independent biological replicates.

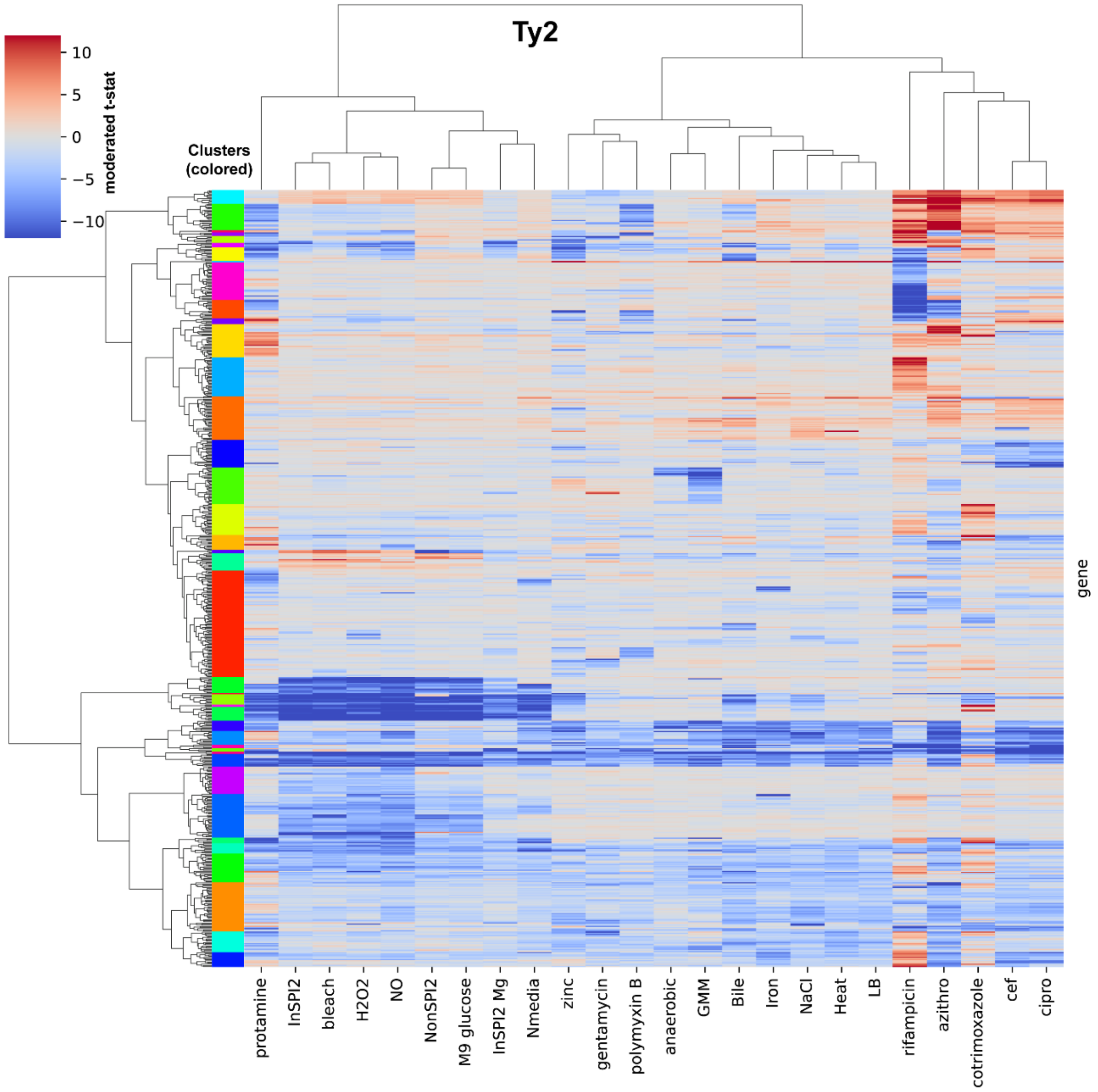

Supplemental Figure 5

Heatmap of genes with significant fitness effects (|moderated t-statistic|>4) from *S.* Typhi Ty2, in which the moderated t-statistic from each gene is plotted across all 24 *in vitro* plate-based conditions. Each gene is an individual row, and each condition is an individual column. Pearson's correlation coefficient was used to calculate the similarity in fitness between pair of genes, and agglomerative clustering was used to cluster genes with more similar fitness effects. This hierarchical clustering was then visualized using dendrograms. Different clusters are highlighted in a series of colors to the left of the heatmap; a full list of genes in these clusters can be found in **Supplemental Data 5**). This heatmap was generated from the average of two independent biological replicates.

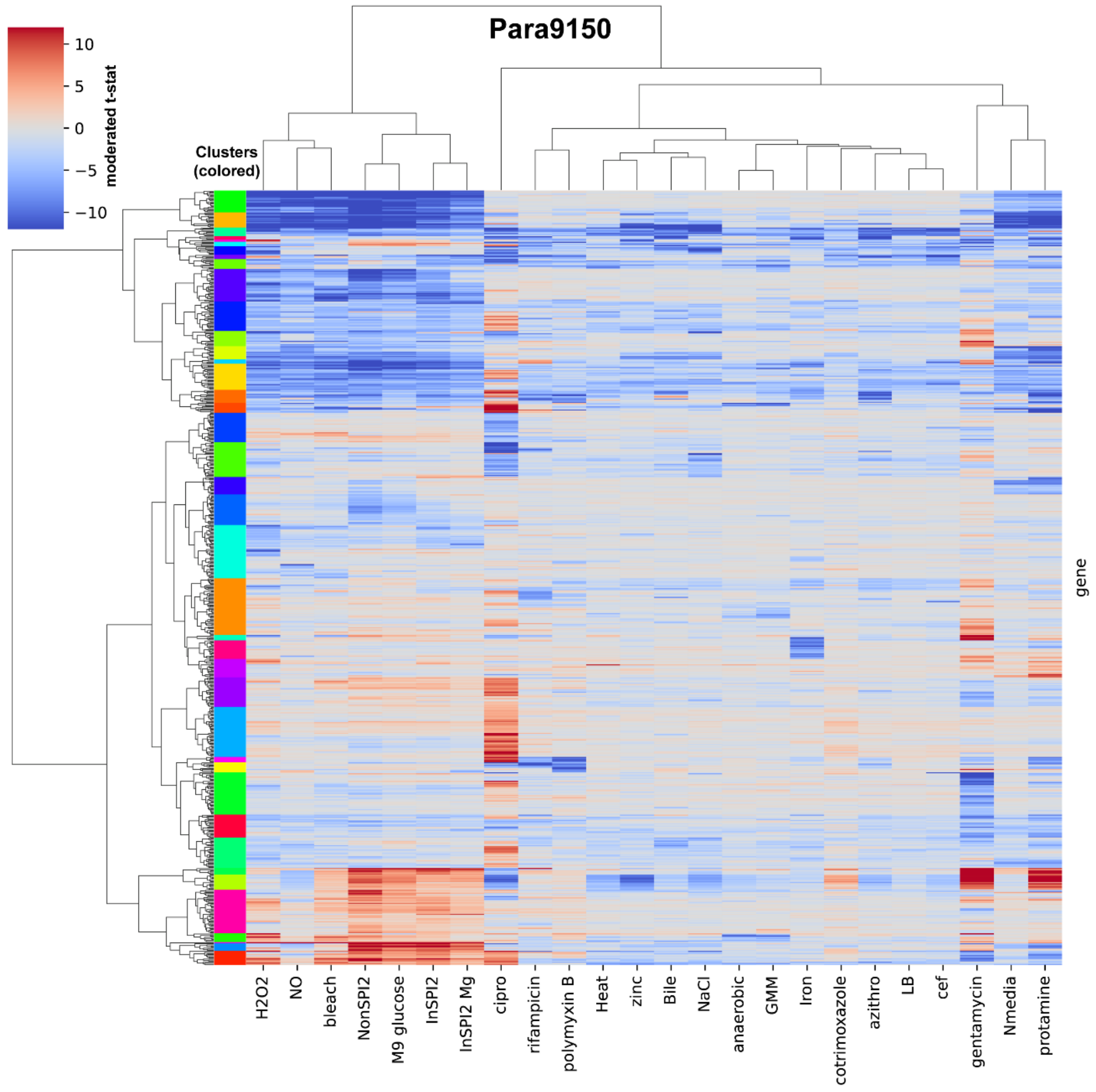

Supplemental Figure 6

Heatmap of genes with significant fitness effects (|moderated t-statistic|>4) from *S.* Paratyphi A 9150, in which the moderated t-statistic from each gene is plotted across all 24 *in vitro* plate-based conditions. Each gene is an individual row, and each condition is an individual column. Pearson's correlation coefficient was used to calculate the similarity in fitness between pair of genes, and agglomerative clustering was used to cluster genes with more similar fitness effects. This hierarchical clustering was then visualized using dendrograms. Different clusters are highlighted in a series of colors to the left of the heatmap; a full list of genes in these clusters can be found in **Supplemental Data 5**). This heatmap was generated from the average of two independent biological replicates.

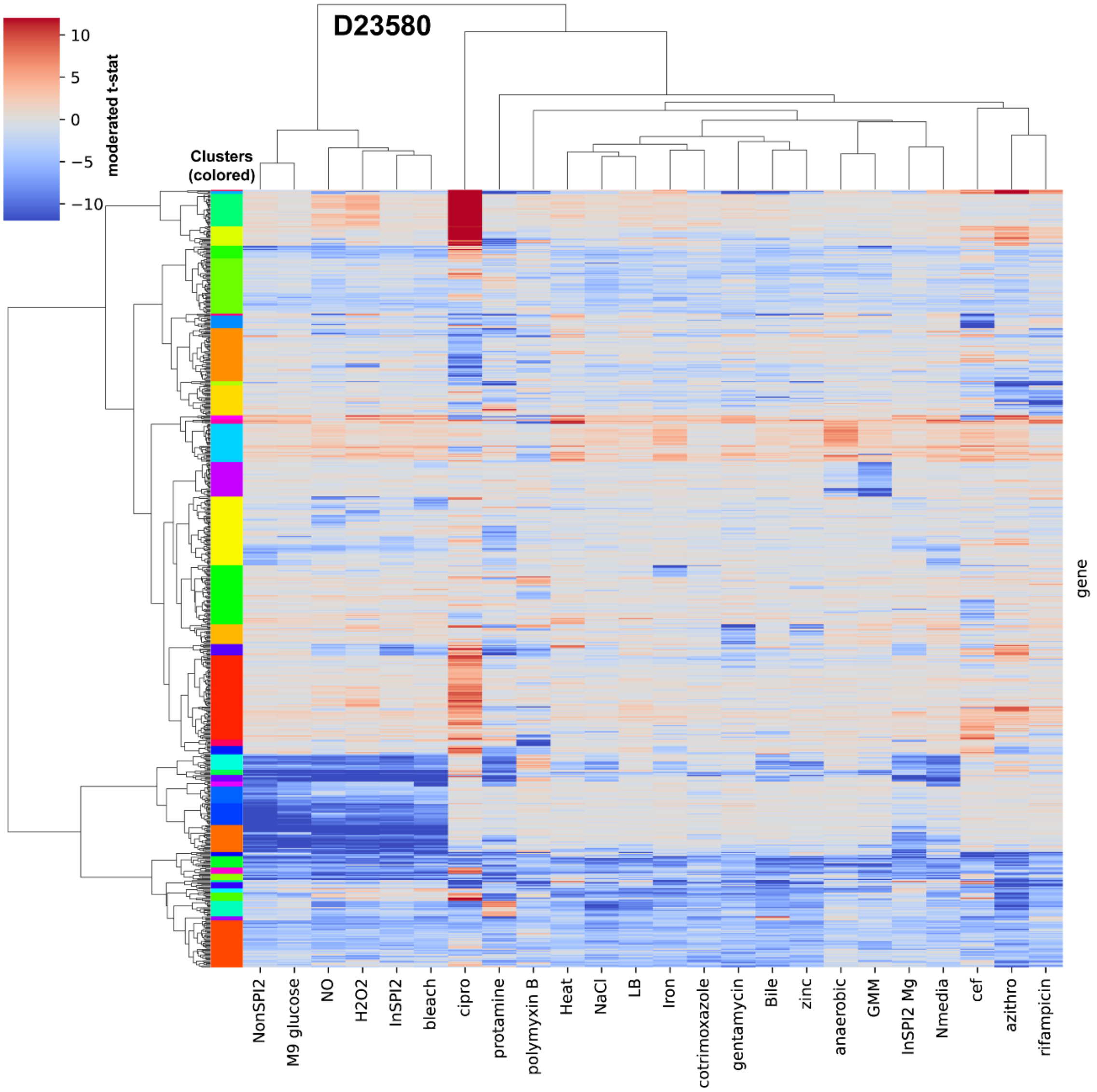

Supplemental Figure 7

Heatmap of genes with significant fitness effects (|moderated t-statistic|>4) from *S.* Typhimurium D23580, in which the moderated t-statistic from each gene is plotted across all 24 *in vitro* plate-based conditions. Each gene is an individual row, and each condition is an individual column. Pearson's correlation coefficient was used to calculate the similarity in fitness between pair of genes, and agglomerative clustering was used to cluster genes with more similar fitness effects. This hierarchical clustering was then visualized using dendrograms. Different clusters are highlighted in a series of colors to the left of the heatmap; a full list of genes in these clusters can be found in **Supplemental Data 5**). This heatmap was generated from the average of two independent biological replicates.

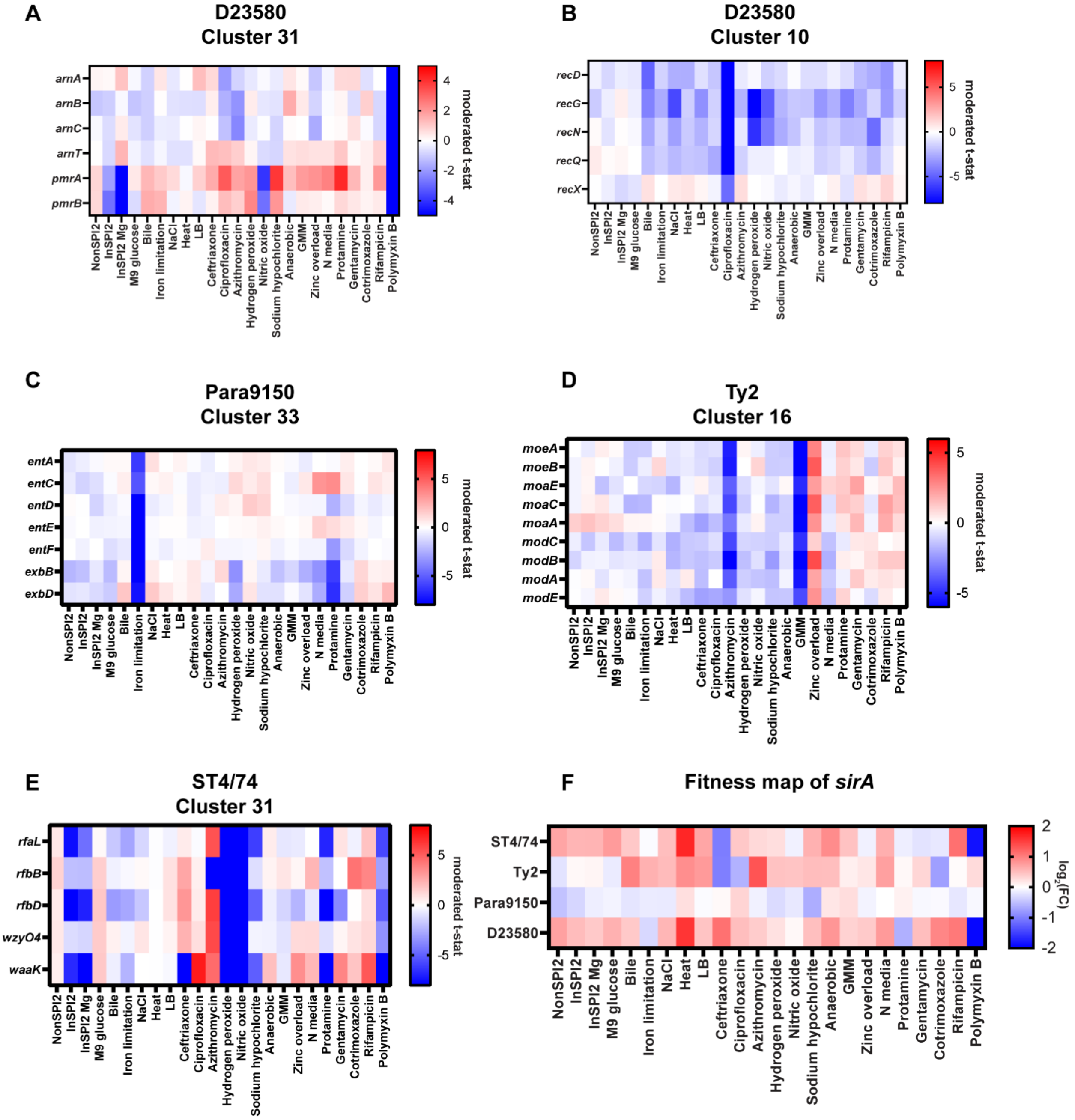

Supplemental Figure 8

A) Heatmap of genes in cluster #31 from D23580**,** comprised of genes in the *arn* LPS-modification operon and the two-component signaling system *pmrAB*. Color gradient is derived from the moderated t-statistic from each condition in the Rb-Tn-seq experiments. **B)** Heatmap of genes in cluster #10 from D23580**,** comprised of genes involved in DNA repair. Color gradient is derived

from the moderated t-statistic from each condition in the Rb-Tn-seq experiments. **C)** Heatmap of genes in cluster #33 from Para9150**,** comprised of genes involved in iron acquisition. Color gradient is derived from the moderated t-statistic from each condition in the Rb-Tn-seq experiments. **D)** Heatmap of genes in cluster #16 from Ty2 comprised of genes involved in molybdenum metabolism. Color gradient is derived from the moderated t-statistic from each condition in the Rb-Tn-seq experiments. **E)** Heatmap of genes in cluster #31 from ST4/74**,** comprised of genes involved in LPS synthesis. Color gradient is derived from the moderated t-statistic from each condition in the Rb-Tn-seq experiments. **F)** Heatmap showing the fitness values of Tn insertions in *sirA* across 24 stress conditions for all four *Salmonella* serovars. Color gradient is derived from the $\log_2$(fitness) from each condition in the Rb-Tn-seq experiments. All fitness values on these heatmaps were generated from the average of two independent biological replicates.

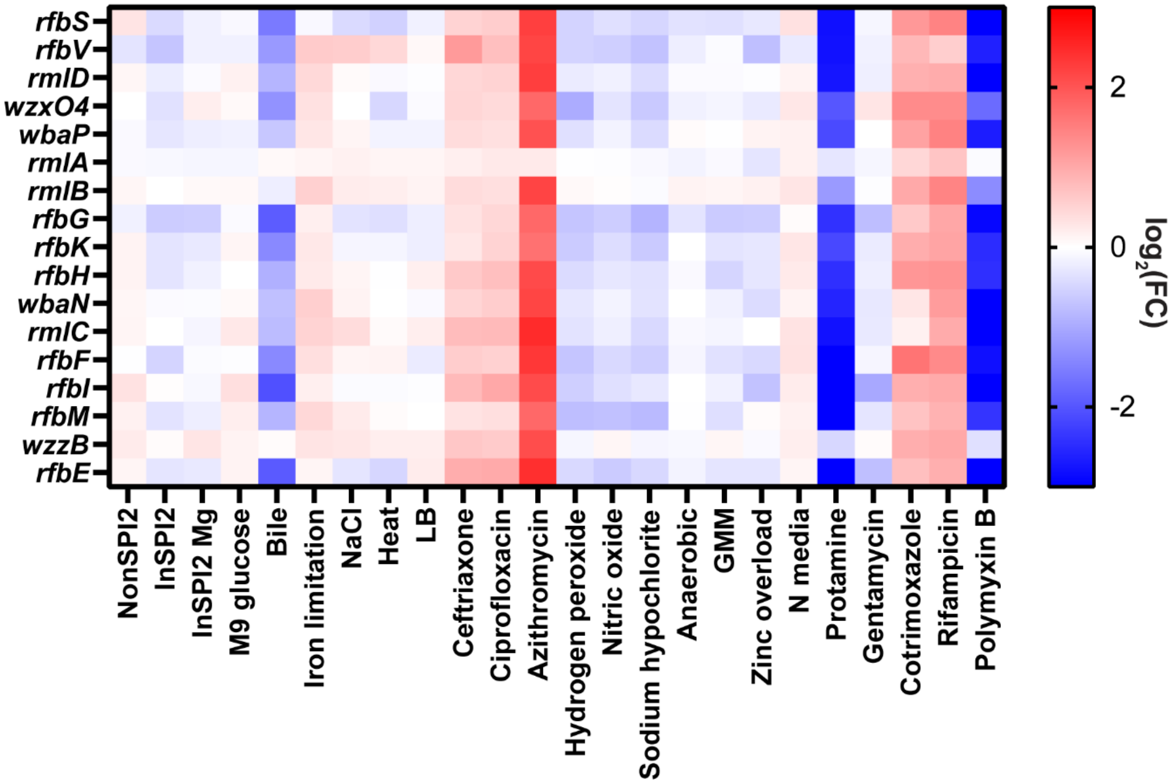

Supplemental Figure 9

Heatmap of genes that comprise the sub-network of LPS-synthesis genes shown in **Fig. 2E.** Color gradient is derived from the $\log_2$(fitness) from each condition in the Rb-Tn-seq experiments. All fitness values on these heatmaps were generated from the average of two independent biological replicates.

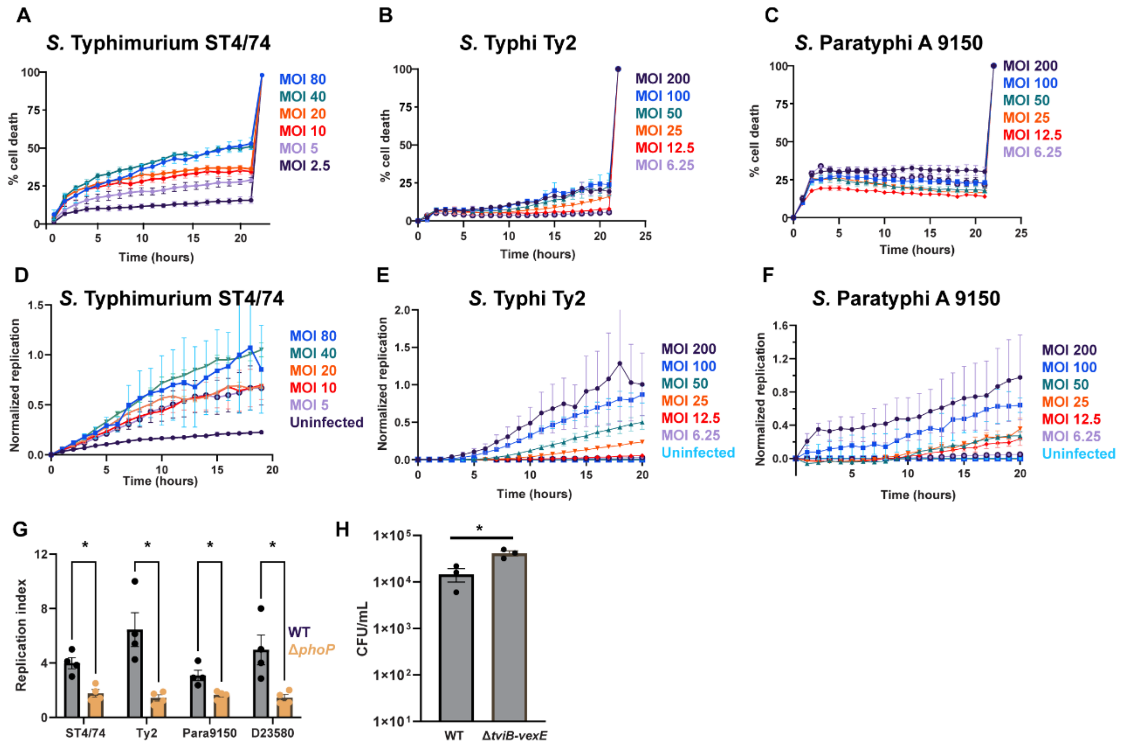

Supplemental Figure 10

A) Cell death assay with *S.* Typhimurium and 20 nM Sytox Green. THP-1s were infected with different MOIs of *S.* Typhimurium ST4/74, with MOI 80 in blue, MOI 40 in green, MOI 20 in orange, MOI 10 in red, MOI 5 in lavender, and MOI 2.5 in black. 10% Triton X-100 was added at 20 hours to lyse all cells, which served as the control for 100% cell death. **B-C)** Cell death assay with *S.* Typhi (**B**) and *S.* Paratyphi A (**C**) and 20 nM Sytox Green. THP-1s were infected with different MOIs of *S.* Typhi Ty2 and *S.* Paratyphi A 9150, with MOI 200 in black, MOI 100 in blue, MOI 50 in green, MOI 25 in orange, MOI 12.5 in red, and MOI 6.25 in lavender. 10% Triton X-100 was added at 20 hours to lyse all cells, which served as the control for 100% cell death. For Sytox Green experiments (**A-C)**, each point and error bar indicates the mean ± SEM of the % of cell death and is taken from one representative experiment with triplicate samples for each condition. **D)** Incucyte-based replication assay for *S.* Typhimurium ST4/74 using the fluorescent dilution pFCcGI plasmid, in which intracellular replication is calculated as the ratio of red to green fluorescence. THP-1s were infected with different MOIs of *S.* Typhimurium ST4/74, with MOI 80 in blue, MOI 40 in green, MOI 20 in orange, MOI 10 in red, MOI 5 in lavender, and uninfected in black. **E-F)** Incucyte-based replication assay for *S.* Typhi Ty2 (**E**) and *S.* Paratyphi A 9150 (**F**) using the fluorescent dilution pFCcGI plasmid, in which intracellular replication is calculated as the ratio of red to green fluorescence. THP-1s were infected with different MOIs of *S.* Typhi Ty2

and *S.* Paratyphi A 9150, with MOI 80 in blue, MOI 40 in green, MOI 20 in orange, MOI 10 in red, MOI 5 in lavender, and uninfected in teal. For replication experiments (**D-F)**, each point and error bar indicates the mean ± SEM of the normalized intracellular replication (red/green ratio), and is taken from one representative experiment with triplicate samples for each condition. **G)** Replication index of intracellular WT (black) and *ΔphoP* (orange) bacterial counts of *S.* Typhimurium ST4/74 (p=0.004), *S.* Typhi Ty2 (p=0.007), *S.* Paratyphi A 9150 (p=0.012), and *S.* Typhimurium D23580 (p=0.02), defined as the ratio of recovered CFU at 20 hours vs 2 hours post-infection. An MOI of 10 was used for *S.* Typhimurium ST4/74 and D23580, and an MOI of 100 was used for *S.* Typhi Ty2 and *S.* Paratyphi A 9150. **H)** Intracellular Ty2^{WT} and Ty2$^{\Delta \textit{tviB-vexE}}$ bacterial counts recovered after 30-minute uptake assay in THP-1 macrophages (p=0.018). For these macrophage CFU plots (**G,H**), bars indicate the mean ± SEM of recovered bacterial CFU with individual values shown; each value is combined from n=4 **(G)** or n=3 **(H)** biologically independent experiments. Significance was calculated using a two-tailed t-test, with * indicating p<0.05.

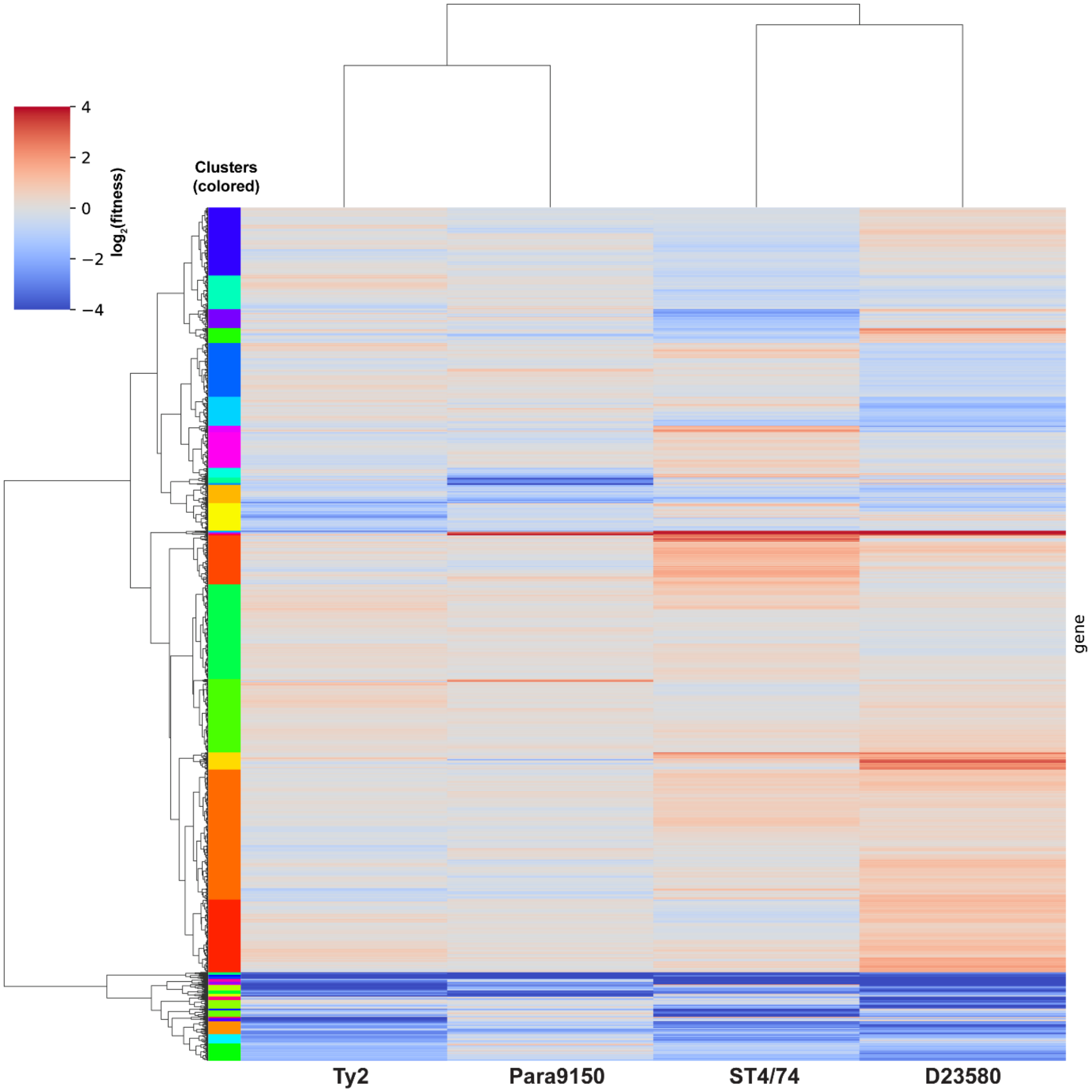

Supplemental Figure 11

Heatmap of genes with significant fitness effects ($p<0.05$ from DEseq2 analysis) from all four isolates, in which the $\log_2$(fitness) from each gene during macrophage infection is plotted. Each gene is an individual row, and each serovar is an individual column. Pearson's correlation coefficient was used to calculate the similarity in fitness between pair of genes, and agglomerative clustering was used to cluster genes with more similar fitness effects. This hierarchical clustering was then visualized using dendrograms. Different clusters are highlighted in a series of colors to the left of the heatmap; a full list of genes in these clusters can be found in **Sup. Table 7**).

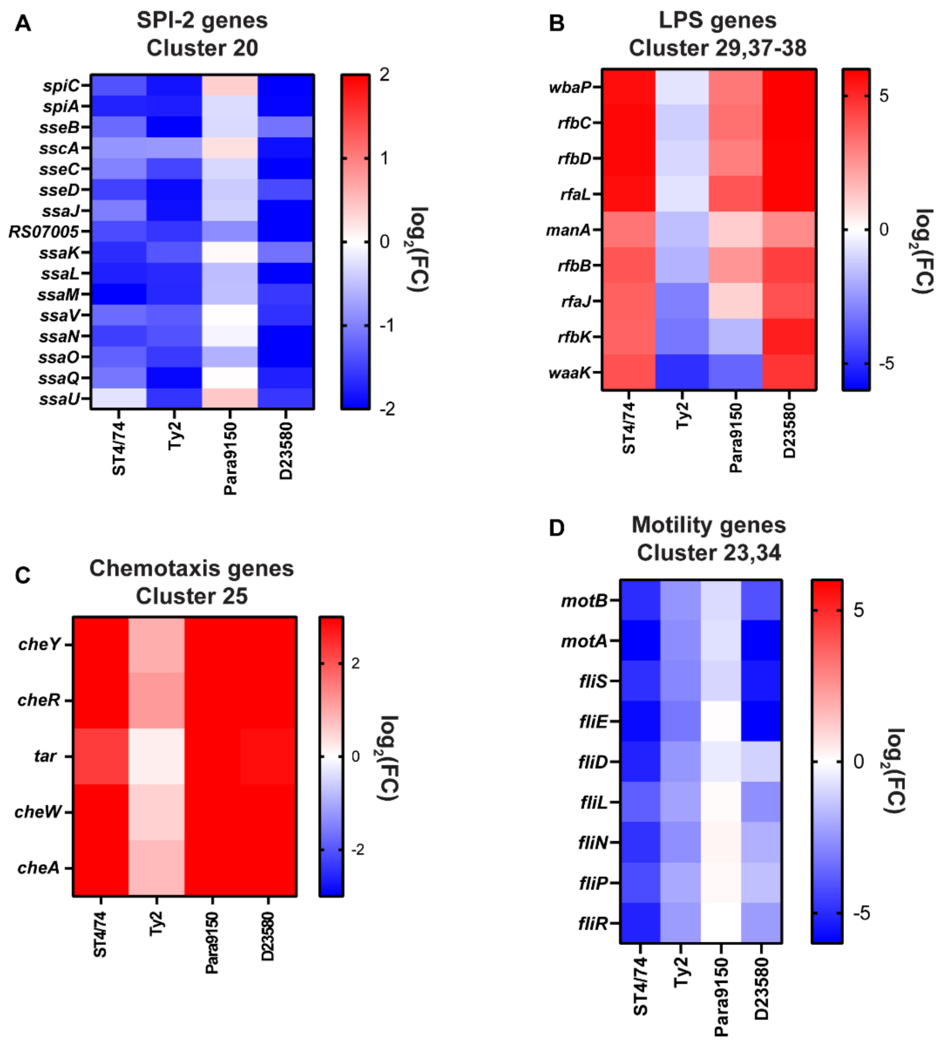

Supplemental Figure 12

A) Heatmap of genes in cluster #20 (see **Sup. Table 7),** comprised of SPI-2 related genes. Color gradient is derived from the log_2(fitness) from each condition in the Rb-Tn-seq experiments. **B)** Heatmap of genes in cluster #29, and #37-38 (see **Sup. Table 7),** comprised of genes involved in LPS synthesis and modification. Color gradient is derived from the log_2(fitness) from each condition in the Rb-Tn-seq experiments. **C)** Heatmap of genes in cluster #25 (see **Sup. Table 7),** comprised of genes involved in chemotaxis. Color gradient is derived from the log_2(fitness) from each condition in the Rb-Tn-seq experiments. **D)** Heatmap of genes in cluster #23, 34 (see **Sup. Table 7),** comprised of genes involved in motility. Color gradient is derived from the log_2(fitness) from each condition in the Rb-Tn-seq experiments.

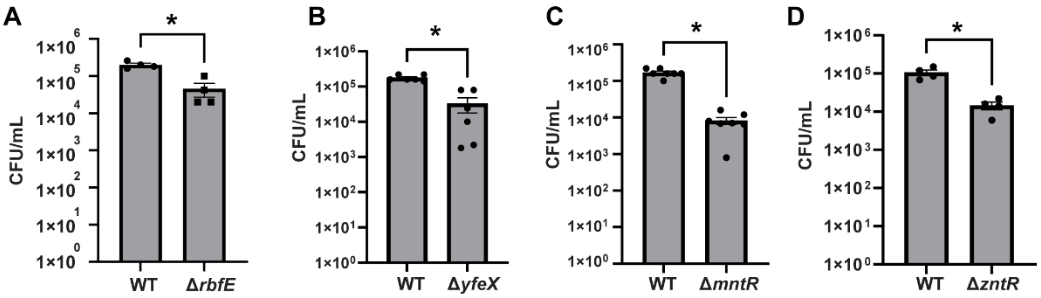

Supplemental Figure 13

A) Intracellular Ty2^{WT} and Ty2$^{\Delta rbfE}$ bacterial counts recovered after 5 hours in LPS-activated THP-1 macrophages (p=0.00169), derived from n=4 independent biological experiments. **B)** Intracellular Ty2^{WT} and Ty2$^{\Delta yfeX}$ bacterial counts recovered after 5 hours in LPS-activated THP-1 macrophages (p=4.03*10^{-5}), derived from n=6 independent biological experiments. **C)** Intracellular Ty2^{WT} and Ty2$^{\Delta mntR}$ bacterial counts recovered after 5 hours in LPS-activated THP-1 macrophages (p=2.95*10^{-7}), derived from n=7 independent biological experiments. **D)** Intracellular Ty2^{WT} and Ty2$^{\Delta zntR}$ bacterial counts recovered after 5 hours in LPS-activated THP-1 macrophages (p=0.00259), derived from n=4 independent biological experiments. For these macrophage CFU plots (**A,B,C,D**), bars indicate the mean ± SEM of recovered bacterial CFU with individual values shown. Significance was calculated using a two-tailed t-test, with * indicating p<0.05.

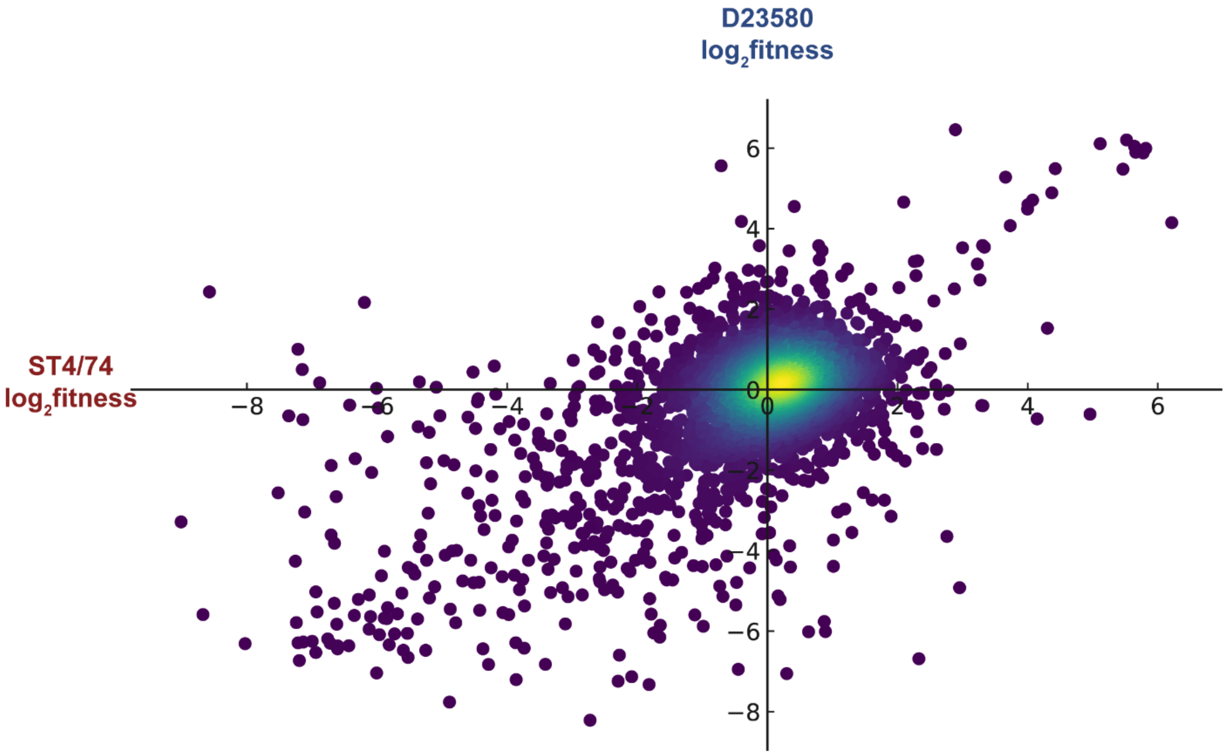

Supplemental Figure 14

Correlation of gene fitness changes between the *S.* Typhimurium ST4/74 and *S.* Typhimurium D23580 from Rb-Tn-seq experiments in macrophages. Each dot represents an individual gene, with the R=0.6 correlation value indicated. Data is derived from the average of three independent biological replicates. Data is shown as a density plot, where areas of increased density are more yellow.

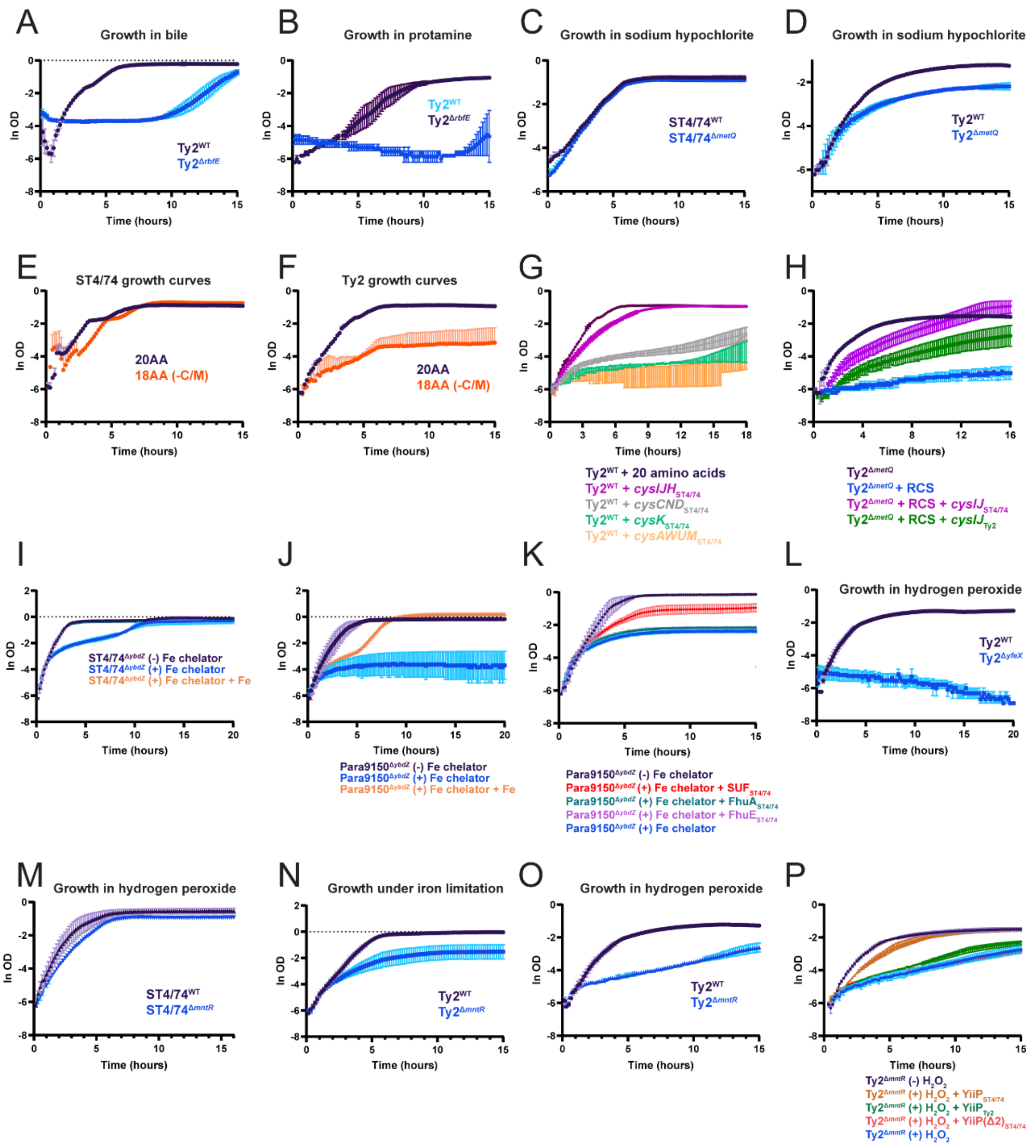

Supplemental Figure 15

Natural-log transformed growth curves of all growth curves found in the main figures. **A** corresponds to Fig. 2G, **B** corresponds to Fig. 2H, **C** corresponds to Fig. 3C, **D** corresponds to Fig. 3D, **E** corresponds to Fig. 3E, **F** corresponds to Fig. 3F, **G** corresponds to Fig. 3G, **H** corresponds to Fig. 3I, **I** corresponds to Fig. 3J, **J** corresponds to Fig. 4D, **K** corresponds to Fig. 4E, **L** corresponds to Fig. 4F, **L** corresponds to Fig. 5E, **M** corresponds to Fig. 6B, **N** corresponds to Fig. 6C, **O** corresponds to Fig. 6D, **P** corresponds to Fig. 6G.

Supplementary Figure 16

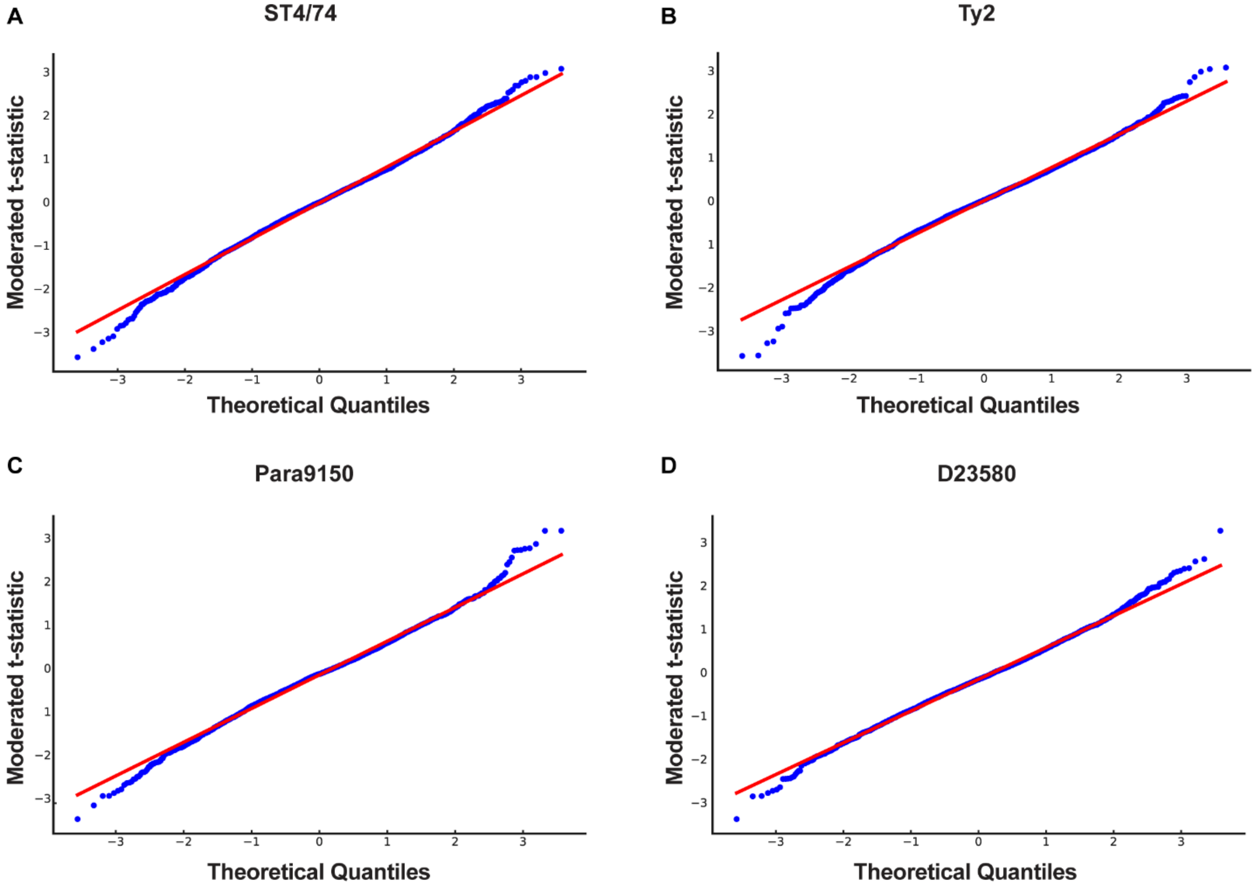

Supplemental Figure 16

Quartile-quartile (QQ) plots, showing the fit of the moderated t-statistic to a normal distribution using replicate time-zero samples, in which no significant changes in fitness are expected. ST4/74 data is shown in **A,** Ty2 data is shown in **B,** Para9150 data is shown in **C,** and D23580 data is shown in **D.**

Supplemental Notes

Supplementary Note 1

We identified 427 to 476 putative essential genes for each serovar**.** Approximately 65% of these essential genes are shared among all 4 serovars, but ~1/3 of these essential genes had serovar-specific phenotypes. For example, IgaA is essential in Typhimurium ST4/74 & D23580[1] but is not essential in Typhi Ty2 or Paratyphi A 9150. Given the relationship between IgaA and the Rcs system[1], this may suggest that Rcs systems are likely mutated in Typhi/Paratyphi A. The alternative sigma factor RpoE and its accessory factors DegS and RseP are essential only in Typhi Ty2, suggesting a unique role in *S.* Typhi. For example, *rpoE* mutations are not tolerated when periplasmic glucans or O-antigen genes are altered[2], so the unique encoding of *rfbE* in Typhi and/or inactivating mutations in *mdoG/H* may underlie this effect. Genes related to iron-sulfur cluster assembly (e.g. *hscA, hscB, fdx, iscU*) are essential in Paratyphi A 9150 but not in Typhimurium ST4/74, indicating alterations in iron metabolism in Paratyphi A (for example, inactivating mutations in the SUF operon, as we later show in Figure 4).

Supplementary Note 2

~15-20% of each network connection includes at least one hypothetical gene. For example, *cpxR* fitness in Typhimurium ST4/74 and D23580 strongly correlated with a hypothetical gene (RS16480/STM_3341 in ST4/74, r=0.96 and RS16305/STMMW_31471 in D23580, r=0.9). Given the association of CpxR with envelope stress[3], this uncharacterized gene may play a role in the CpxR-mediated envelope response pathway in Typhimurium. Similarly, the uncharacterized gene RS03310/t0654 exhibited strong correlation (r>0.9) with 24 amino acid metabolism genes in Typhi (**Supplementary Data 10)**, suggesting its involvement in the transcriptional regulation of amino acid metabolism in typhoidal *Salmonella.* Finally, many prophage-related genes in S. Typhimurium D23580 contained within the ST64B prophage (eg, RS10205, RS10230, RS10265, etc., many of which are genes of unknown function) cluster together with each other and with genes involved in DNA replication & repair. These genes have very strong fitness effects in the presence of DNA-damaging stressors, including hydrogen peroxide and ciprofloxacin. This data suggests that specific prophages in *Salmonella* are being induced under infection-related stress and sub-lethal doses of antibiotics, which may have intriguing effects on the physiology of this pathogen.

Supplementary Note References

1. Cano, D. A., Domínguez-Bernal, G., Tierrez, A., Portillo, F. G. & Casadesús, J. Regulation of Capsule Synthesis and Cell Motility in *Salmonella enterica* by the Essential Gene *igaA*. *Genetics* **162**, 1513–1523 (2002).
2. Amar, A., Pezzoni, M., Pizarro, R. A. & Costa, C. S. New envelope stress factors involved in σE activation and conditional lethality of rpoE mutations in Salmonella enterica. *Microbiology (Reading)* **164**, 1293–1307 (2018).
3. Raivio, T. L., Leblanc, S. K. D. & Price, N. L. The Escherichia coli Cpx envelope stress response regulates genes of diverse function that impact antibiotic resistance and membrane integrity. *J Bacteriol* **195**, 2755–67 (2013).
