# Supplementary material for: High-throughput fitness experiments reveal specific vulnerabilities of human-adapted Salmonella during stress and infection
Source: Nat Genet. 2024 Jun 3;56(6):1288–99. doi: 10.1038/s41588-024-01779-7 (PMC11176087; doi:10.1038/s41588-024-01779-7)
Supplement: Supplementary file 2 — Reporting Summary [file 41588_2024_1779_MOESM2_ESM.pdf]

nature portfolio

Corresponding author(s): Denise M. Monack

Last updated by author(s): 03/28/2024

Reporting Summary

Nature Portfolio wishes to improve the reproducibility of the work that we publish. This form provides structure for consistency and transparency in reporting. For further information on Nature Portfolio policies, see our Editorial Policies and the Editorial Policy Checklist.

Statistics

For all statistical analyses, confirm that the following items are present in the figure legend, table legend, main text, or Methods section.

n/a	Confirmed	
☐	☒	The exact sample size (*n*) for each experimental group/condition, given as a discrete number and unit of measurement
☐	☒	A statement on whether measurements were taken from distinct samples or whether the same sample was measured repeatedly
☐	☒	The statistical test(s) used AND whether they are one- or two-sided<br>*Only common tests should be described solely by name; describe more complex techniques in the Methods section.*
☒	☐	A description of all covariates tested
☐	☒	A description of any assumptions or corrections, such as tests of normality and adjustment for multiple comparisons
☐	☒	A full description of the statistical parameters including central tendency (e.g. means) or other basic estimates (e.g. regression coefficient) AND variation (e.g. standard deviation) or associated estimates of uncertainty (e.g. confidence intervals)
☐	☒	For null hypothesis testing, the test statistic (e.g. *F*, *t*, *r*) with confidence intervals, effect sizes, degrees of freedom and *P* value noted<br>*Give P values as exact values whenever suitable.*
☒	☐	For Bayesian analysis, information on the choice of priors and Markov chain Monte Carlo settings
☒	☐	For hierarchical and complex designs, identification of the appropriate level for tests and full reporting of outcomes
☐	☒	Estimates of effect sizes (e.g. Cohen's *d*, Pearson's *r*), indicating how they were calculated

Our web collection on statistics for biologists contains articles on many of the points above.

Software and code

Policy information about availability of computer code

Data collection	No special software was used in this study
Data analysis	All data was analyzed using published methods; for Rb-Tn-seq fitness experiments, we used the pipeline described in Wetmore et al., 2015 in mbio. For SAFE analysis, we used the pipeline described in Leshchiner et al., 2022 in Nature Communications. For these analyses, we used perl (Ubuntu for windows 20.04.3 LTS), Cytoscape (3.9.1), and jupyter notebook (6.4.12).

For manuscripts utilizing custom algorithms or software that are central to the research but not yet described in published literature, software must be made available to editors and reviewers. We strongly encourage code deposition in a community repository (e.g. GitHub). See the Nature Portfolio guidelines for submitting code & software for further information.

Data

Policy information about availability of data

All manuscripts must include a data availability statement. This statement should provide the following information, where applicable:

- Accession codes, unique identifiers, or web links for publicly available datasets
- A description of any restrictions on data availability
- For clinical datasets or third party data, please ensure that the statement adheres to our policy

All sequencing data is publicly accessible at NCBI GEO with accession numbers (GSE261860, GSE261867, GSE261873, GSE261757, GSE261749, GSE261214). All data from Rb-Tn-seq is also searchable with our user-interactive website, SalcomFit, at https://bioinf.gen.tcd.ie/cgi-bin/salcomfit.pl.

Research involving human participants, their data, or biological material

Policy information about studies with human participants or human data. See also policy information about sex, gender (identity/presentation), and sexual orientation and race, ethnicity and racism.

Reporting on sex and gender	This is not relevant to our study.
Reporting on race, ethnicity, or other socially relevant groupings	This is not relevant to our study.
Population characteristics	This is not relevant to our study.
Recruitment	This is not relevant to our study.
Ethics oversight	This is not relevant to our study.

Note that full information on the approval of the study protocol must also be provided in the manuscript.

Field-specific reporting

Please select the one below that is the best fit for your research. If you are not sure, read the appropriate sections before making your selection.

☒ Life sciences ☐ Behavioural & social sciences ☐ Ecological, evolutionary & environmental sciences

For a reference copy of the document with all sections, see nature.com/documents/nr-reporting-summary-flat.pdf

Life sciences study design

All studies must disclose on these points even when the disclosure is negative.

Sample size	All Rb-Tn-seq experiments were done in biological duplicate, and correlation values were very high (generally R>0.8). All other experiments were done with at least 3 independent biological replicates. Exact sample sizes are reported below each figure, in the caption. Sample sizes were not predetermined. However, our replicate numbers were in line with the field (n at least 3 for all experiments). In addition, we note that both our Rb-Tn-seq results and individual growth curves/mutant validations were all highly reproducible, suggesting that our sample sizes were sufficient.
Data exclusions	No data was excluded.
Replication	We quantified the correlation value (R) for all Rb-Tn-seq experiments and found that they are very high (R~0.85 across all experiments on average), indicating that the data is reproducible. All other experiments were repeated at least 3 independent times, with exact numbers reported below each figure, in the caption.
Randomization	This is not applicable to our study, because all bacterial strains/samples were subjected to the same set of conditions.
Blinding	This is not applicable to our study, because all bacterial strains/samples were treated in the same way, with the same set of conditions.

Reporting for specific materials, systems and methods

We require information from authors about some types of materials, experimental systems and methods used in many studies. Here, indicate whether each material, system or method listed is relevant to your study. If you are not sure if a list item applies to your research, read the appropriate section before selecting a response.

Materials & experimental systems

n/a	Involved in the study	
☒	☐	Antibodies
☐	☒	Eukaryotic cell lines
☒	☐	Palaeontology and archaeology
☒	☐	Animals and other organisms
☒	☐	Clinical data
☒	☐	Dual use research of concern
☒	☐	Plants

Methods

n/a	Involved in the study	
☒	☐	ChIP-seq
☒	☐	Flow cytometry
☒	☐	MRI-based neuroimaging

Eukaryotic cell lines

Policy information about cell lines and Sex and Gender in Research

Cell line source(s)	ATCC THP-1
Authentication	ATCC authenticated these cells using STR profiling.
Mycoplasma contamination	We confirmed that the cells did not contain mycoplasma contamination using PCR.
Commonly misidentified lines (See ICLAC register)	No commonly misidentified cell lines were used in this study.

Plants

Seed stocks	*Report on the source of all seed stocks or other plant material used. If applicable, state the seed stock centre and catalogue number. If plant specimens were collected from the field, describe the collection location, date and sampling procedures.*
Novel plant genotypes	*Describe the methods by which all novel plant genotypes were produced. This includes those generated by transgenic approaches, gene editing, chemical/radiation-based mutagenesis and hybridization. For transgenic lines, describe the transformation method, the number of independent lines analyzed and the generation upon which experiments were performed. For gene-edited lines, describe the editor used, the endogenous sequence targeted for editing, the targeting guide RNA sequence (if applicable) and how the editor was applied.*
Authentication	*Describe any authentication procedures for each seed stock used or novel genotype generated. Describe any experiments used to assess the effect of a mutation and, where applicable, how potential secondary effects (e.g. second site T-DNA insertions, mosiacism, off-target gene editing) were examined.*
